# Classification of Surface Electromyography Recordings from Young and Older Adults Using CWT Scalograms: A Window-Level Proof-of-Concept Study

**DOI:** 10.3390/diagnostics16213584

**Published:** 2026-10-30

**Authors:** SoHyeon Bang, JinHyoung Jeong, YuYeon Jung

**Affiliations:** 1 Department of Electronic and Communication Engineering, Catholic Kwandong University, Gangneung-si 25601, Republic of Korea; thgus7572@cku.ac.kr; 2 Department of Healthcare Management, Catholic Kwandong University, 24 Beomil-ro 579 Beongil, Gangneung-si 25601, Republic of Korea; wlsgud0201@cku.ac.kr; 3 Department of Dental Hygiene, College of Medical Science, Konyang University, 158 Gwanjeodong-ro, Seo-gu, Daejeon 35365, Republic of Korea

**Keywords:** age-group classification, surface electromyography, continuous wavelet transform, deep learning, ResNet-18, time–frequency analysis

## Abstract

**Background/Objectives:** Age-related changes in skeletal muscle involve alterations in both muscle structure and neuromuscular function. Surface electromyography (sEMG) provides a non-invasive method for assessing muscle electrical activity. This study aimed to investigate whether time–frequency representations of sEMG signals combined with deep learning could classify recordings obtained from healthy young and older adults, without assuming that the discriminative features necessarily reflect age-related neuromuscular changes. **Methods:** sEMG signals were experimentally collected from 60 healthy adults, including 30 young and 30 older adults. The resulting continuous wavelet transform (CWT) scalogram images were randomly divided into training and test sets at the image-window level using an 80:20 ratio. Consequently, windows from the same participant could appear in both sets. Signals were filtered, segmented into overlapping windows, and converted into CWT scalogram images for classification using ResNet-18. **Results:** At the window level, the CWT-ResNet model achieved an accuracy of 78.98%, an area under the receiver operating characteristic curve (AUC) of 0.8589, a sensitivity of 71.34%, a specificity of 85.06%, and a balanced accuracy of 78.20%. Muscle-specific analyses showed window-level classification accuracies ranging from 78.57% to 79.89% across all three muscle sites. However, the observed classification performance may have been influenced by differences in movement timing, active and resting periods, anthropometric characteristics, and participant overlap between the training and test sets. These potential influences were not isolated through additional control analyses. **Conclusions:** CWT-based time–frequency representations of sEMG combined with ResNet-18 demonstrated preliminary feasibility for classifying sEMG recordings obtained from young and older adults under the revised preprocessing and image-window-level evaluation conditions. The findings do not establish that the model specifically detects age-related neuromuscular changes. Participant-independent validation and analyses controlling for task execution and anthropometric characteristics are required.

## 1. Introduction

### 1.1. Research Background and Necessity

#### 1.1.1. The Social Crisis of an Aging Society and Sarcopenia

Advancements in modern medicine and improvements in living standards have extended the average human lifespan, but this has simultaneously presented a massive social challenge known as ‘aging’. In this context, sarcopenia is defined as an independent disease that threatens health in old age, going beyond the simple process of aging [1].

Sarcopenia is a syndrome characterized by a decrease in skeletal muscle mass accompanied by a decline in muscle strength and physical performance. This goes beyond simply slowing walking speed and leads to falls, fractures, exacerbation of chronic diseases, and a significant increase in mortality [2]. In Korea, sarcopenia is classified under the KCD-8 code M62.5, reflecting its recognition as a clinically important condition requiring appropriate assessment and management. In particular, the importance of ‘timely diagnosis’ is being emphasized more than ever because sarcopenia can be reversibly recovered if appropriate exercise prescription and nutrition are provided in the early stages of onset [3].

#### 1.1.2. Technical and Economic Limitations of Existing Diagnostic Technologies

Current consensus frameworks for sarcopenia, including the Asian Working Group for Sarcopenia (AWGS) 2019 and the European Working Group on Sarcopenia in Older People 2 (EWGSOP2), emphasize that sarcopenia assessment should not rely on muscle mass alone but should also consider muscle strength and physical performance [4,5]. Imaging-based methods such as dual-energy X-ray absorptiometry (DXA), magnetic resonance imaging (MRI), and computed tomography (CT) can provide quantitative information on muscle mass and structural characteristics, but they do not independently establish a diagnosis of sarcopenia.

Despite their usefulness for assessing muscle quantity and morphology, these imaging techniques have practical limitations for large-scale or repeated assessment. MRI and CT involve relatively high equipment and operational costs and generally require specialized clinical facilities [6]. Moreover, imaging-based assessments primarily characterize structural aspects of muscle and do not directly evaluate muscle strength, physical performance, or neuromuscular activation. Therefore, complementary functional assessment approaches may provide additional information that cannot be obtained from muscle quantity alone [7].

### 1.2. Purpose of Study

#### 1.2.1. Surface Electromyography as a Tool for Investigating Muscle Activity

Surface electromyography (sEMG) provides a non-invasive approach for recording electrical activity generated during muscle contraction. Unlike imaging-based assessments that primarily characterize muscle structure, sEMG enables the investigation of muscle activation patterns during voluntary movement [8]. Accordingly, sEMG can provide complementary information about muscle electrical activity under defined experimental conditions.

In the present study, sEMG was used to investigate whether recordings obtained from healthy young and older adults contained distinguishable time–frequency patterns. The objective was to evaluate the preliminary feasibility of classifying CWT scalogram windows according to the age group from which they originated, rather than to establish a diagnostic method for sarcopenia or directly identify specific physiological mechanisms of muscle aging.

#### 1.2.2. Limitations of 1D Signal Analysis and the Introduction of 2D CWT Analysis

Traditional sEMG analysis has primarily utilized one-dimensional statistical indicators such as amplitude in the time domain (RMS) or mean frequency in the frequency domain (MNF). However, the electromyogram signals of patients with sarcopenia are very complex, non-stationary, and dynamically change with contraction intensity and time [9]. It is difficult to precisely identify changes in muscle fiber types or subtle disruptions in neural firing patterns hidden within the signal using only simple one-dimensional indicators.

To overcome these limitations, this study introduces Continuous Wavelet Transform (CWT). CWT enables the simultaneous analysis of time and frequency components, visualizing which frequency components occurred at a specific point in time in the form of a two-dimensional image (Scalogram) [10]. This transformation preserves time-varying signal characteristics by representing one-dimensional sEMG data as two-dimensional time–frequency information.

#### 1.2.3. Deep-Learning-Based Classification of Age-Group-Associated sEMG Patterns

This study combined two-dimensional time–frequency representations obtained using the continuous wavelet transform (CWT) with a convolutional neural network (CNN). Previous research has demonstrated that CNN-based architectures can automatically extract discriminative features from sEMG signals for classification tasks [11]. The primary objective was to investigate whether sEMG-derived CWT scalogram windows obtained from healthy young and older adults could be classified according to their source age group under an image-window-level evaluation framework.

One-dimensional sEMG recordings were transformed into CWT scalograms and analyzed using a ResNet-18 CNN. We hypothesized that the resulting time–frequency representations would contain patterns associated with age-group labels that could be learned by the model. However, successful classification was not assumed to demonstrate that these patterns specifically represented age-related neuromuscular alterations.

We also examined descriptive window-level classification performance across the medial gastrocnemius, rectus femoris, and erector spinae muscle sites and used Grad-CAM to visualize image regions contributing to individual model predictions. Accordingly, the study was designed as a preliminary proof-of-concept investigation of age-group-associated information in sEMG recordings, without claiming participant-independent generalization, physiological validation, or diagnostic utility for sarcopenia.

### 1.3. Related Research and Theories

#### 1.3.1. Electromyography Signals and Muscle Aging

Aging is accompanied by structural and functional alterations in the neuromuscular system, including changes in motor unit properties, neuromuscular transmission, and neural input to skeletal muscles. Recent evidence suggests that these neuromuscular alterations can be detected even in older individuals without clinically diagnosed sarcopenia, indicating that changes in neuromuscular function may emerge before the clinical manifestation of sarcopenia [12,13].

sEMG provides a non-invasive method for evaluating neuromuscular activity and has demonstrated potential for detecting age-related changes in muscle function. High-density sEMG studies have shown that electromyographic indices can distinguish age-related differences in muscle function and identify alterations associated with muscle aging [14]. Furthermore, recent systematic evidence suggests that sEMG may provide useful information for the assessment of muscle function in older adults, although methodological standardization and further clinical validation remain necessary [7].

These findings support the use of sEMG as a physiological signal for characterizing age-related neuromuscular changes and provide a rationale for investigating whether such changes can be captured using advanced signal-processing and deep-learning approaches.

#### 1.3.2. Age-Related Changes in Motor Unit Properties and Neural Input

Aging is associated with progressive alterations in the neuromuscular system, including changes in motor unit properties, neuromuscular junction (NMJ) stability, and neural input to skeletal muscle [15,16]. These alterations may contribute to age-related declines in muscle force and neuromuscular function and are not necessarily explained by reductions in muscle mass alone.

Recent electrophysiological evidence has demonstrated age-related differences in motor unit behavior. High-density sEMG studies have shown reduced persistent inward currents and common synaptic inputs to vastus lateralis motor units in older adults compared with younger adults, indicating alterations in neural input to muscle with aging [12]. Such changes provide evidence that aging affects not only muscle morphology but also the neural mechanisms involved in muscle activation.

Because sEMG reflects the collective electrical activity generated during muscle activation, age-related alterations in neural input and motor unit behavior may influence the temporal and spectral characteristics of recorded sEMG signals. Therefore, analysis of sEMG in the time–frequency domain may provide a useful approach for characterizing complex neuromuscular changes associated with aging without assuming that a single electromyographic feature represents a specific physiological mechanism.

#### 1.3.3. Age-Related Changes in the Frequency Characteristics of sEMG Signals

The frequency characteristics of sEMG signals are influenced by multiple physiological factors, including muscle fiber conduction velocity, motor unit recruitment and discharge behavior, and muscle fatigue. Accordingly, frequency-domain measures such as mean frequency (MNF) and median frequency (MDF) have been widely used to characterize changes in neuromuscular function. However, these spectral measures should not be interpreted as direct indicators of a single physiological mechanism because the observed sEMG spectrum reflects the combined effects of multiple neuromuscular processes [17].

In a study involving individuals with chronic low back pain, Ebenbichler et al. reported age-related differences in the temporal evolution of back-muscle sEMG spectral characteristics during repeated contractions, with younger individuals showing more pronounced frequency changes than older individuals [17]. These findings indicate that aging may influence not only the overall spectral distribution of sEMG but also its time-dependent response during sustained or repeated muscle activation.

More recent investigations have further demonstrated the potential value of spectral sEMG features for characterizing neuromuscular function in older adults. Matsumoto et al. used frequency-domain analysis to examine muscle fiber recruitment characteristics during voluntary contraction in trained older adults, supporting the use of spectral sEMG measures for investigating age-related muscle function [18].

Collectively, these findings suggest that age-related neuromuscular alterations may be reflected in complex and time-varying changes in the frequency characteristics of sEMG signals. Therefore, rather than relying solely on individual summary measures such as MNF or MDF, analysis methods that preserve both temporal and frequency information may provide a more comprehensive representation of age-related sEMG patterns.

### 1.4. Deep-Learning-Based Biosignal Analysis

Conventional analysis of physiological signals, including electromyography (EMG), electrocardiography (ECG), and electroencephalography (EEG), has commonly relied on handcrafted features extracted from the time, frequency, or time–frequency domains. These features are subsequently used as inputs to conventional machine-learning classifiers such as support vector machines and random forests. Although handcrafted feature-based approaches remain useful, their performance can depend strongly on feature selection and prior domain knowledge, and predefined features may not fully represent complex patterns within physiological signals. Deep learning provides an alternative approach by learning hierarchical representations directly from raw signals or transformed signal representations. In particular, convolutional neural networks (CNNs) have been increasingly applied to biomedical signals because convolutional filters can automatically learn discriminative local and spatial features from structured input data [19]. For sEMG analysis, deep-learning approaches can therefore reduce dependence on manually selected features and enable the investigation of complex signal representations relevant to classification tasks [20,21].

#### 1.4.1. Application of Biosignals to Convolutional Neural Networks (CNNs)

Convolutional neural networks (CNNs) have been increasingly applied to sEMG analysis because of their ability to learn discriminative features from complex signal representations. Two general strategies can be considered for CNN-based sEMG analysis. The first applies one-dimensional CNN architectures directly to segmented sEMG signals, allowing temporal patterns to be learned without requiring manual feature extraction. Recent studies have demonstrated that CNN-based architectures can effectively classify sEMG signals by learning relevant representations directly from segmented signal data [10].

An alternative strategy is to transform one-dimensional sEMG signals into two-dimensional representations and subsequently apply image-based CNN architectures. Time–frequency transformations are particularly relevant for sEMG because the signal is non-stationary and its spectral characteristics can vary over time. Wavelet-based transformations can represent these temporal and spectral characteristics simultaneously, producing two-dimensional representations that can be analyzed using CNNs [22].

These approaches provide the methodological basis for combining time–frequency analysis with deep learning. In the present study, CWT was used to transform segmented sEMG signals into two-dimensional scalograms, and a ResNet-18 CNN was subsequently employed to learn discriminative time–frequency patterns associated with age-group classification.

#### 1.4.2. Subject-Independent Evaluation and Generalization

A major challenge in deep-learning-based sEMG analysis is the substantial inter-subject variability of signal characteristics. Differences in physiological characteristics, electrode placement, and recording conditions can affect the distribution of sEMG signals and may reduce model performance when applied to previously unseen individuals. Therefore, subject-independent evaluation, in which participants used for testing are excluded from the training dataset, is important for assessing the generalizability of sEMG-based classification models.

These findings highlight the importance of evaluating sEMG-based deep-learning models under subject-independent conditions. Although transfer learning can provide an additional strategy for improving cross-subject generalization [22], rigorous separation of participants between training and evaluation datasets remains necessary to determine whether a model can generalize to previously unseen individuals.

In the present study, the original evaluation employed random image-window-level partitioning rather than participant-independent partitioning. Consequently, overlapping windows originating from the same participant could appear in both training and test sets, potentially leading to optimistic performance estimates. Therefore, the reported classification performance should be interpreted as preliminary window-level results rather than evidence of generalization to previously unseen participants.

## 2. Methods

### 2.1. Experimental Design and Data Collection

**Figure 1:**
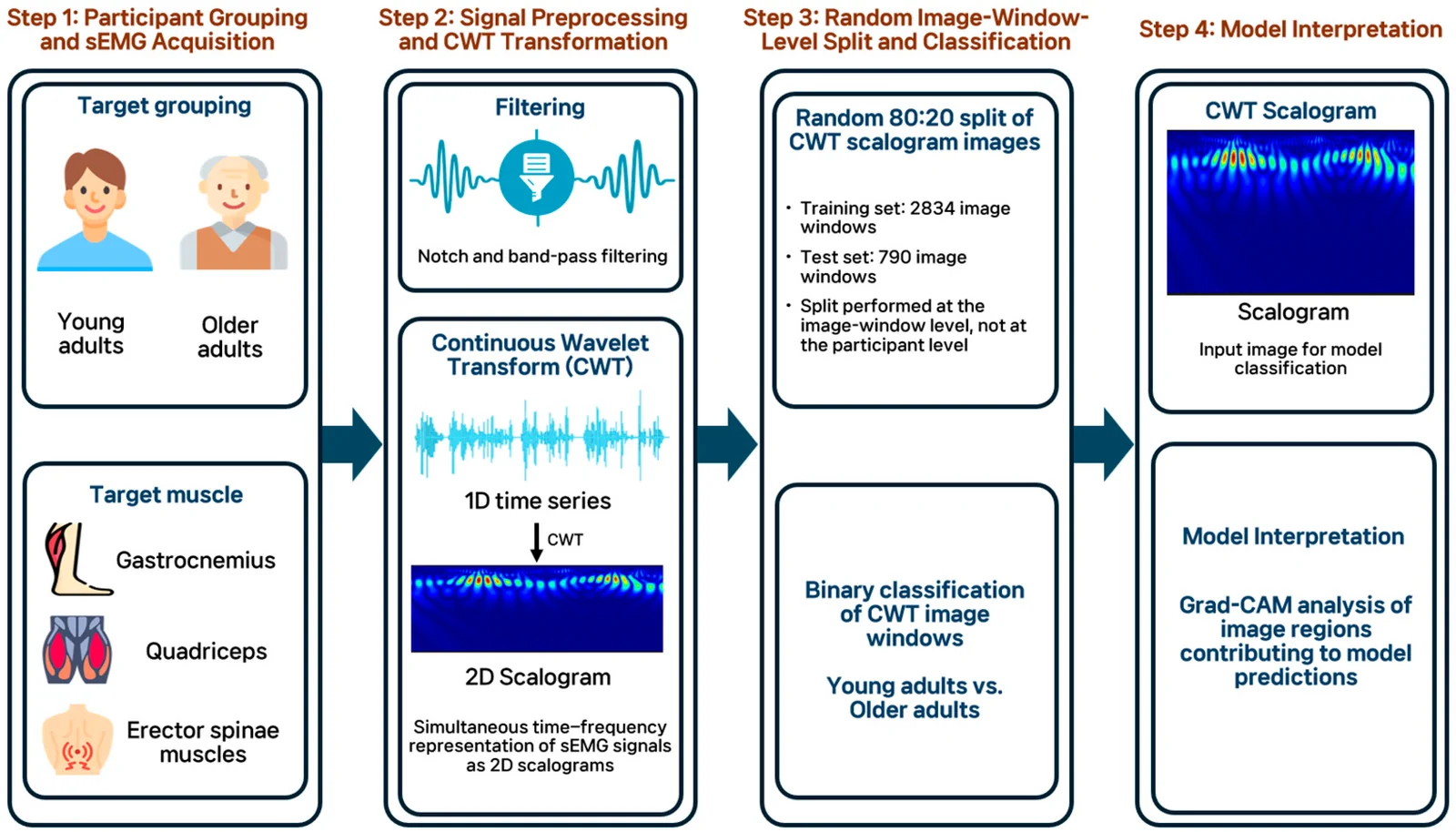
Overview of the study workflow. Following sEMG acquisition, signal preprocessing, and CWT scalogram generation, the resulting images were randomly divided into training and test subsets at the image-window level using an 80:20 ratio. ResNet-18 was used for age-group classification, and Grad-CAM was applied to visualize image regions contributing to model predictions. Participant-independent partitioning was not performed.

Figure 1 summarizes the overall experimental workflow, including participant grouping, sEMG acquisition, signal preprocessing, CWT scalogram generation, random image-window-level dataset partitioning, classification, and model interpretation.

#### 2.1.1. Participant Selection and Grouping

Eligible young adults were aged 20–29 years, whereas older adults were aged 65 years or older. In the enrolled sample, the observed age range was 21–29 years in the young group and 65–81 years in the older group. The young group consisted of 15 men and 15 women, and the older group consisted of 14 men and 16 women. The demographic characteristics of the participants are summarized in Table 1.

Participants were recruited at Catholic Kwandong University, Republic of Korea. Older adults were recruited from participants enrolled in an educational program for older adults conducted at the university. Data collection began on 25 October 2025, and continued through May 2026. Health eligibility was assessed based on self-reported medical history, including the presence of known musculoskeletal or neurological disorders that could affect performance of the experimental tasks or acquisition of sEMG signals.

Participants were classified according to chronological age rather than clinical sarcopenia status. Therefore, the older group should not be interpreted as a clinically diagnosed sarcopenia group. Grip strength, gait speed, and SARC-F scores were collected from both age groups to characterize participants’ physical function, and the descriptive results are summarized in Table 1. These measures were not used as inputs to the deep-learning model or included in the classification analysis. The purpose of age-based grouping was to investigate whether sEMG recordings contained distinguishable age-group-associated signal patterns.

#### 2.1.2. sEMG Measurement Sites

sEMG signals were recorded from three muscle sites: the rectus femoris, medial gastrocnemius, and erector spinae. These muscles were selected to represent lower-limb and trunk muscle activity involved in weight-bearing, locomotion, and postural control. For the rectus femoris, electrodes were placed over the anterior central region of the thigh. For the medial gastrocnemius, electrodes were placed over the medial aspect of the posterior calf. For the erector spinae, electrodes were placed over the paraspinal muscle region of the lower trunk according to the predefined measurement protocol. The rectus femoris contributes primarily to knee extension, the medial gastrocnemius contributes to ankle plantar flexion and propulsion during gait, and the erector spinae contributes to trunk extension and postural stabilization. By recording sEMG signals from these three anatomically and functionally distinct muscle sites, this study aimed to determine whether age-group-associated differences in sEMG patterns were observed consistently across multiple muscle sites rather than being confined to a single site.

#### 2.1.3. sEMG Data Acquisition Protocol

sEMG signals were acquired using a PhysioLab EMG system equipped with a PSL-iEMG2 two-channel electromyography amplifier module at a sampling frequency of 2000 Hz. According to the manufacturer’s specifications, the amplifier has a nominal gain of 500 V/V, a 10-Hz high-pass filter, a 200-Hz low-pass filter, and a selectable 50/60-Hz notch filter. Commercially available 3M surface electrode patches were used for bipolar sEMG recording. Electrodes were placed over the predefined muscle regions, with their positions adjusted according to the anatomical location and morphology of each participant’s target muscles. The skin surface was prepared before electrode attachment according to laboratory protocol. A fixed inter-electrode distance was not enforced, and the center-to-center distance between electrodes was not measured or systematically recorded. Bilateral measurements were performed for the medial gastrocnemius, rectus femoris, and erector spinae.

All participants performed the same predefined muscle-specific tasks under standardized instructions. For the medial gastrocnemius, participants performed repeated heel raises in a standing position while lightly supporting themselves against a wall, with the contralateral leg flexed to reduce its contribution to the movement. For the rectus femoris, participants were seated with the knee positioned at approximately 45° and performed repeated knee-extension efforts against manual resistance applied by the examiner. For the erector spinae, participants performed repeated trunk-extension movements using a back-extension apparatus. Each task was repeated five times, and sEMG was recorded for 3 s during each repetition. Although the recording protocol specified five 3-s repetitions for each muscle and recording side in both age groups, the retained CWT image windows were not equally distributed between the two groups. The test subset contained 709 image windows, comprising 314 windows from older adults (44.29%) and 395 windows from young adults (55.71%). These counts represent image windows rather than independent participants because the dataset was randomly partitioned at the image-window level. The available test-set counts do not establish the specific source of this imbalance. The anatomical regions targeted for electrode placement, body positions, and task instructions were consistent across both age groups, although the exact electrode positions were adjusted according to individual muscle morphology.

Task cadence and movement speed were not externally controlled using a metronome or force-feedback system. Participants performed the tasks at a self-selected pace within the standardized movement protocol. Force, torque, and percentage of maximal voluntary contraction (%MVC) were not recorded. The acquired sEMG signals were subsequently processed using the filtering, segmentation, and time–frequency transformation procedures described in Section 2.2. Channel-wise normalization was subsequently applied to the scalogram image tensors before ResNet-18 classification.

#### 2.1.4. Dataset Construction

The collected sEMG recordings were organized according to participant group and muscle region. Recordings from the medial gastrocnemius, rectus femoris, and erector spinae muscles, including bilateral measurements, were retained for subsequent analyses. The recordings were processed and segmented into overlapping windows, which were subsequently transformed into CWT scalogram images.

The reorganized dataset comprised 300 source sEMG text files (150 from young adults and 150 from older adults). Applying the updated preprocessing procedure yielded 3543 CWT image windows, including 1885 from the young group and 1658 from the older group.

Although both age groups comprised 30 participants and 150 source recordings each, the total retained recording duration was 1052.25 s for young adults and 939.85 s for older adults. Applying identical 1-s windows with a 50% overlap resulted in 1885 and 1658 CWT windows, respectively. Thus, the difference in window counts was associated with variation in the lengths of retained recordings rather than unequal numbers of source files. The distribution of source recordings and CWT windows by muscle site and recording side is presented in Table 1.

The resulting image dataset was partitioned as detailed in Section 2.3.1.

The demographic and anthropometric characteristics of the study participants are summarized in Table 2. Body mass index (BMI) was calculated individually for each participant by dividing body weight (kg) by the square of height (m^2^). Height, body weight, and BMI are presented as the mean ± standard deviation (SD). Between-group differences in these anthropometric characteristics were evaluated using two-sided Welch’s independent-sample *t*-tests, with statistical significance set at *p* < 0.05.

Values are presented as the mean ± standard deviation. Grip strength, gait speed, and SARC-F were assessed in both age groups (*n* = 30 per group) for participant characterization and were not used as inputs to the classification model. Dashes indicate that between-group statistical tests were not performed for these measures.

### 2.2. Data Preprocessing and CWT Conversion

#### 2.2.1. Physiological Signal Filtering

Raw sEMG signals were acquired at a sampling frequency of 2000 Hz. For CWT scalogram generation, a two-stage digital filtering procedure was implemented using SciPy with a sampling-frequency parameter of 2000 Hz. First, an infinite impulse response (IIR) notch filter was designed with a center frequency of 60 Hz and a quality factor (Q) of 30. Subsequently, a fourth-order Butterworth band-pass filter was designed with cutoff frequencies of 20 and 450 Hz. Both filters were applied using forward–backward zero-phase filtering (‘filtfilt’).

#### 2.2.2. Window Segmentation and Data Partitioning

Following filtering, the sEMG recordings were segmented into 1-s windows consisting of 2000 samples with 50% overlap (1000-sample step size) at a sampling frequency of 2000 Hz. No explicit activity detection or contraction-onset detection algorithm was applied, and inactive segments were not specifically removed before segmentation. Consequently, individual windows could contain varying proportions of active contraction, transition periods, and relative rest.

The segmented windows were subsequently transformed into CWT scalograms as described in Section 2.2.3.

Following CWT scalogram generation, the images were partitioned as described in Section 2.3.1.

#### 2.2.3. 2D Imaging of sEMG: CWT

CWT was applied to each segmented sEMG window to generate a two-dimensional time-scale representation [10]. The CWT was implemented using the PyWavelets library with the real-valued Morlet wavelet (morl) as the mother wavelet, without additional user-defined wavelet parameters. Scales ranging from 1 to 127 were used, resulting in 127 scale levels. The mathematical definition of the CWT is expressed as follows.

(1) $$X_{\omega }(a,\;b)=\frac{1}{\sqrt{\left|a\right|}}\;\int_{-\infty }^{\infty }x(t)\varphi^{*}(\frac{t-b}{a})\mathrm{dt}$$

In Equation (1), *a* denotes the scale parameter, and *b* denotes the translation parameter. Each sEMG window contained 2000 samples, producing a CWT coefficient matrix of 127 × 2000, corresponding to scale and sample position, respectively. The sampling-period parameter supplied to the CWT function was 1/2000 s, consistent with the sampling frequency used for signal acquisition and preprocessing.

The absolute values of the CWT coefficients were converted into scalogram images and saved in PNG format using Matplotlib’s ‘imsave’ function with the ‘jet’ colormap. No explicit per-window z-score standardization of the sEMG signal or separate normalization of the CWT coefficients was performed in the CWT image-generation code. Because fixed color limits were not specified, the magnitude values were mapped to colors according to the range of each individual coefficient matrix.

For ResNet-18 classification, the saved scalograms were loaded as three-channel RGB images, resized to 224 × 224 pixels, converted into image tensors, and normalized using the ImageNet channel means [0.485, 0.456, 0.406] and standard deviations [0.229, 0.224, 0.225]. This channel-wise normalization was applied to the image tensors and should be distinguished from amplitude normalization of the original sEMG signals. The scalograms were used for age-group classification; muscle fatigue was not independently assessed.

**Figure 2:**
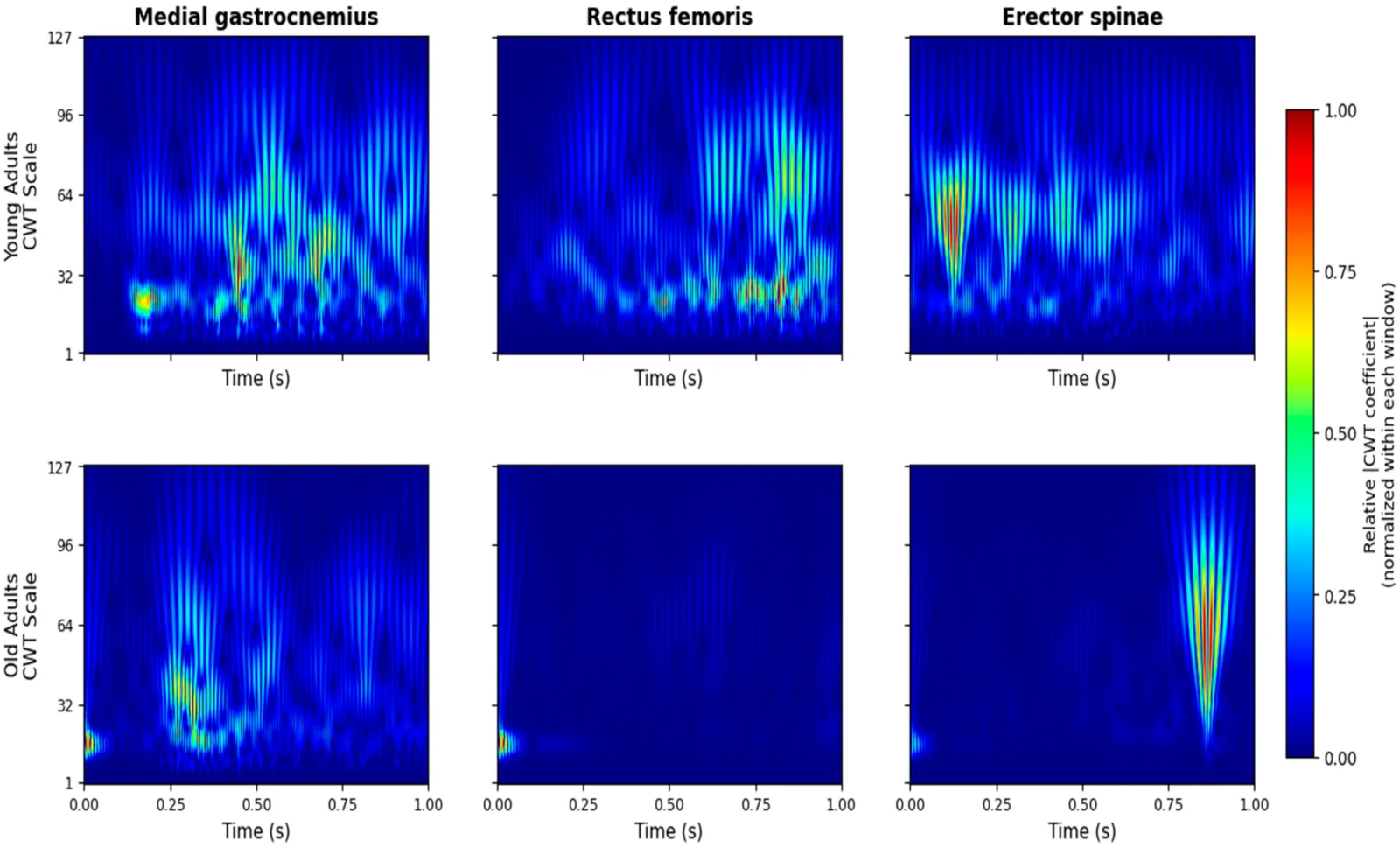
CWT scalograms of sEMG recordings from young and older adults. The upper and lower rows display examples from the young and older groups, respectively. The columns represent the medial gastrocnemius, rectus femoris, and erector spinae muscles. Each panel displays the first 1-s window of the first valid recording selected in alphabetical file order for the corresponding age group and muscle site. The horizontal axis represents time (0–1 s), and the vertical axis represents the CWT scale (1–127). Color intensity indicates the relative magnitude of CWT coefficients normalized independently within each window (0–1); therefore, absolute amplitudes cannot be compared across panels.

Representative CWT scalograms obtained from recordings of young and older adults are shown in Figure 2.

### 2.3. Deep-Learning Model Design

#### 2.3.1. Image-Window-Level Data Partitioning

The complete CWT image dataset was divided into training (80%) and test (20%) subsets using the PyTorch ‘random_split’ function with a fixed split seed of 42. Partitioning was performed at the image-window level after CWT scalogram generation, without grouping by participant or stratification by age group or sex. Consequently, windows from the same participant, including overlapping windows and recordings from different muscle sites, could appear in both subsets.

The split seed of 42 was specified for dataset partitioning; the provided training code did not establish deterministic initialization and training across all computational operations. No independent validation subset was created. Test accuracy was evaluated after each of the 20 training epochs, and the checkpoint with the highest test accuracy was selected for final evaluation, as detailed in Section 2.3.2. Therefore, the reported results represent the original image-window-level evaluation and do not establish generalization to previously unseen participants.

#### 2.3.2. Deep-Learning Architecture: ResNet-18 Architecture and Residual Learning

To analyze the two-dimensional CWT scalograms, the ResNet-18 (Residual Network-18) architecture, a deep convolutional neural network, was employed [23]. ResNet uses residual learning with skip connections to facilitate gradient propagation and address optimization difficulties associated with increasing network depth. The operation of a residual block is defined as follows:

*y* = *F*(*x*, {*W_i_*}) + *x* (2)

Here, (*x*) denotes the input, (*F*(*x*,{*W_i_*})) represents the residual mapping learned by the convolutional layers with parameters ({*W_i_*}), and (*y*) denotes the output of the residual block. By adding the input directly to the residual mapping, the network facilitates stable gradient propagation and learns discriminative spatial patterns from CWT scalograms for age-group classification.

The ResNet-18 model was initialized using ImageNet-pretrained weights (IMAGENET1K_V1), and its final fully connected layer was replaced with a two-output classification layer for young and older adults. Input images were resized to 224 × 224 pixels, converted into three-channel RGB tensors, and normalized using ImageNet channel means of [0.485, 0.456, 0.406] and standard deviations of [0.229, 0.224, 0.225], as described in Section 2.2.3.

The model was trained for 20 epochs with a batch size of 16 using the Adam optimizer, a learning rate of 0.0001, and cross-entropy loss. A constant learning rate was used without a learning-rate scheduler or early stopping criterion. No explicit class weighting, data augmentation, or class-balanced sampling was implemented. The training dataset was shuffled at each epoch, whereas the test dataset was evaluated without shuffling.

Dataset partitioning is described in Section 2.3.1. Model accuracy was evaluated on the test subset after each training epoch, and the checkpoint achieving the highest test accuracy was saved and selected for final evaluation. The selected checkpoint achieved a test accuracy of 78.98% and a ROC-AUC of 0.8589. No separate validation dataset was used for checkpoint selection or hyperparameter optimization.

The computational environment was based on Google Colab with Python 3.13.16. The installed software versions included PyTorch 2.11.0+cu130, Torchvision 0.26.0+cu130, NumPy 2.1.3, SciPy 1.16.3, PyWavelets 1.8.0, Matplotlib 3.10.0, scikit-learn 1.6.1, Pillow 11.3.0, and Pandas 2.2.3. The environment was equipped with an NVIDIA Tesla T4 GPU and CUDA 13.0.

#### 2.3.3. Grad-CAM Application for Model Interpretability

In the fields of medicine and biomechanics, it is essential to understand the rationale behind classification results (Young vs. Old) derived by deep-learning models. In this study, the Grad-CAM (Gradient-weighted Class Activation Mapping) algorithm was applied to interpret the black-box characteristics of the model [24]. Activation values and backpropagating gradients from the feature maps generated in the last convolutional layer (layer4[-1]) of the ResNet-18 model were extracted using hooks. Spatial importance was calculated through global average pooling-based weighting operations for the target class (e.g., Old), and only the regions that had a positive influence on the model’s judgment were visualized as activation maps using the ReLU activation function. Through this procedure, the time–frequency regions that contributed to the model’s classification decisions were visualized and qualitatively examined. The Grad-CAM results were used for model interpretation only and were not considered physiological validation of age-related muscle changes. In addition, a single ResNet-18 model was trained using pooled CWT scalograms from all three muscle sites, rather than training separate models for individual muscles. The trained model was subsequently evaluated separately on test windows from the medial gastrocnemius, rectus femoris, and erector spinae to report muscle-specific classification performance.

## 3. Results

We quantitatively evaluated the window-level classification performance of the proposed CWT-ResNet-based age-group classification model using a random image-window-level train–test split. Because the dataset was not partitioned at the participant level, the reported performance does not establish generalization to previously unseen participants. In addition, we examined model performance across three muscle regions with different anatomical characteristics.

### 3.1. Overall Performance Evaluation

The proposed CWT-ResNet model achieved a window-level classification accuracy of 78.98% (560/709) for distinguishing sEMG recordings from young and older adults. Of the 709 test windows, 395 (55.71%) belonged to the young group and 314 (44.29%) belonged to the older group. Consequently, a majority-class classifier that always predicted the young group would achieve an accuracy of 55.71%.

The available CWT image dataset exhibited an imbalanced distribution between age groups, which was reflected in the test set. The number of image windows should not be interpreted as the number of independent observations or as evidence of equivalent recording durations between age groups. Differences in window counts may reflect variation in recording procedures, segmentation, or data availability and require further investigation.

With older adults defined as the positive class, the model achieved a sensitivity of 71.34%, specificity of 85.06%, positive predictive value (PPV) of 79.15%, negative predictive value (NPV) of 78.87%, balanced accuracy of 78.20%, and Matthews correlation coefficient (MCC) of approximately 0.57. The area under the receiver operating characteristic curve (ROC-AUC) was 0.8589. However, these performance metrics were obtained using random image-window-level partitioning rather than participant-independent evaluation. Therefore, they should be interpreted as descriptive results of the revised evaluation rather than evidence of generalization to previously unseen participants.

**Figure 3:**
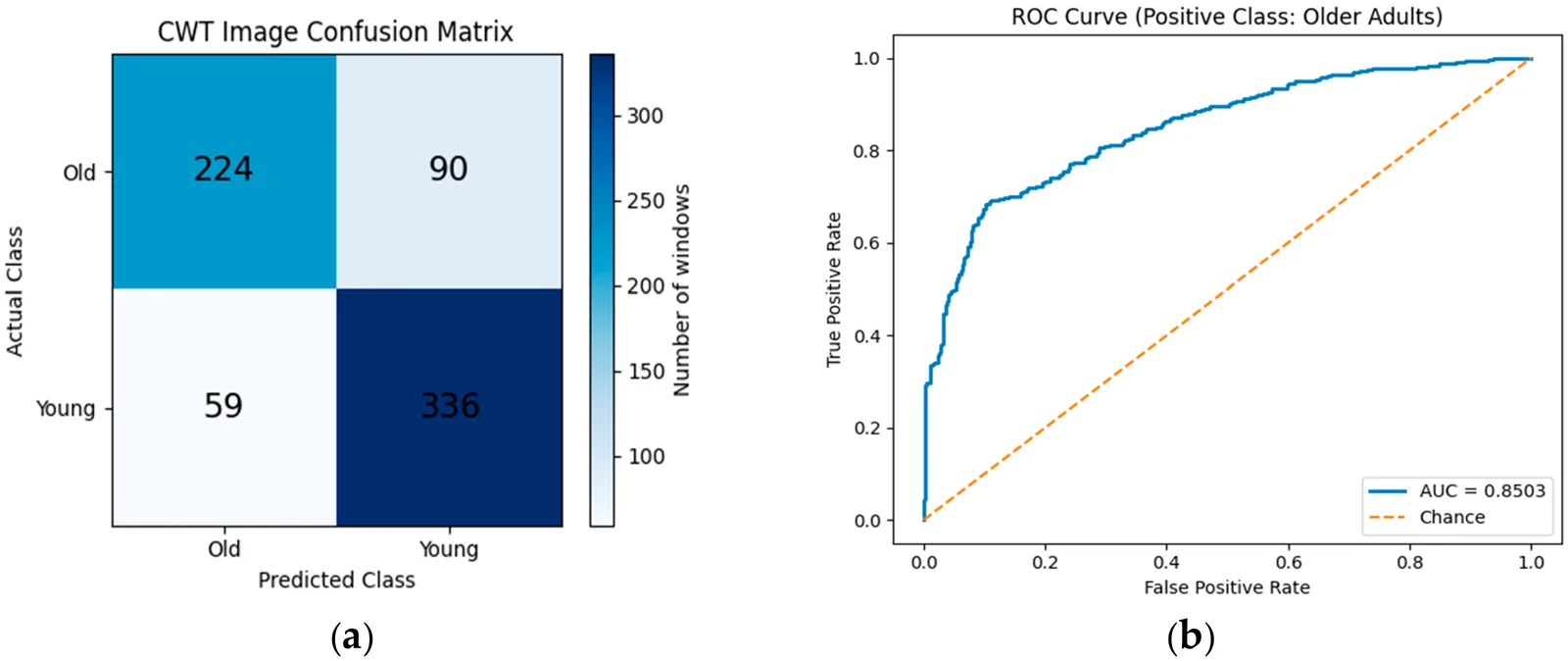
Confusion matrix and ROC curve of the proposed CWT-ResNet model. (**a**) Confusion matrix showing the classification results for the young and older groups at a softmax decision threshold of 0.5. (**b**) Receiver operating characteristic (ROC) curve generated using the predicted probabilities for the older-adult class, with an area under the curve (AUC) of 0.8589. All reported predictions were evaluated at the image-window level, with older adults defined as the positive class.

Figure 3 presents the confusion matrix and ROC curve obtained from the classification model. For binary classification, the predicted class was determined by selecting the class with the higher softmax probability, corresponding to a decision threshold of 0.5 for the older-adult class. The confusion matrix summarizes window-level predictions for the young and older groups, while the ROC curve illustrates classification performance across different decision thresholds. Because the dataset was randomly partitioned at the image-window level, windows originating from the same participants could be included in both training and test subsets. Furthermore, the 50% overlap between adjacent windows introduced additional sample dependence, potentially allowing the model to learn participant-specific patterns and resulting in optimistic performance estimates. Participant-level classification performance and corresponding bootstrap confidence intervals were not calculated. Therefore, further evaluation using participant-independent cross-validation is required to establish the generalizability of the proposed model.

### 3.2. Analysis of Results by Muscle Region

Muscle-specific evaluation was performed by applying the trained CWT-ResNet model to test windows from each muscle site. As shown in Table 3, the window-level classification accuracies were 78.81% (212/269) for the medial gastrocnemius, 78.57% (209/266) for the rectus femoris, and 79.89% (139/174) for the erector spinae. Overall, 560 of 709 test windows were correctly classified.

Although the observed accuracies ranged from 78.57% to 79.89% across the three muscle sites, these results should not be interpreted as demonstrating differences in classification performance between muscles. Muscle-specific confidence intervals and participant-independent performance estimates were not available, and the test windows were not statistically independent at the participant level. Therefore, the present findings provide descriptive window-level classification results rather than evidence of superior performance at any muscle site.

## 4. Discussion

This study investigated a CWT-ResNet-18-based framework for distinguishing sEMG recordings obtained from young and older adults using CWT scalograms. The model demonstrated window-level age-group classification performance under the applied random train–test split. However, because the data were partitioned at the image-window level rather than by participant, the findings should not be interpreted as evidence of participant-independent generalization. This section discusses the observed classification results, their potential physiological relevance, and the methodological limitations of the proposed approach.

### 4.1. Interpretation of Deep-Learning Decision Basis Based on Grad-CAM

**Figure 4:**
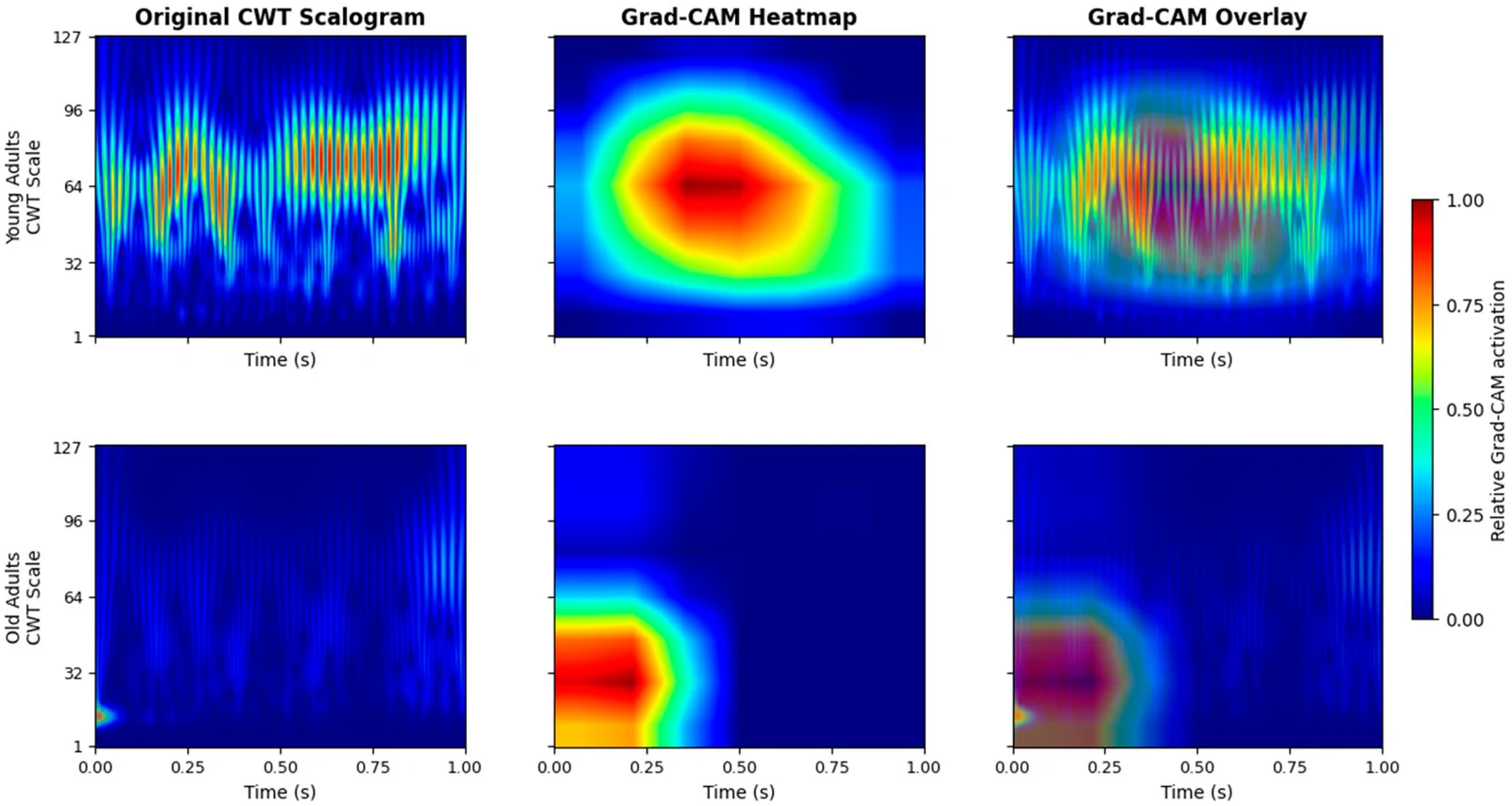
Representative Grad-CAM visualizations generated using the CWT-ResNet-18 model. The upper and lower rows show correctly classified test windows from young and older adults, respectively, selected as the first correctly classified image in alphabetical file-path order within each age group. The left column presents the original CWT scalograms, the middle column shows the Grad-CAM activation maps, and the right column displays the activation maps overlaid on the original scalograms. The horizontal axis represents time (0–1 s), and the vertical axis represents CWT scale (1–127). The color bar represents relative Grad-CAM activation normalized to the range 0–1 for each displayed window. Warmer colors indicate regions with higher relative Grad-CAM activation for the corresponding predicted age-group class. These visualizations illustrate model attention for individual examples and do not establish age-specific physiological differences.

Grad-CAM was applied to visualize the regions of the CWT scalograms that contributed to the ResNet-18 model’s classification decisions. Figure 4 presents representative Grad-CAM visualizations for one young-adult window and one older-adult window. The highlighted regions indicate areas that influenced the model’s predictions for these individual examples.

However, the visualizations are based on selected windows and do not establish consistent differences in attention patterns between age groups. In particular, the present examples do not provide sufficient evidence to conclude that the older-adult group exhibits more localized activation or that the young-adult group exhibits broader activation across the scalograms. Accordingly, the Grad-CAM findings are interpreted as illustrative examples of model attention rather than quantitative evidence of group-specific time–frequency characteristics.

In the older-adult example, the maximal Grad-CAM activation did not necessarily coincide with the highest-energy region of the original scalogram, suggesting that model attention may also have been influenced by the temporal placement of activity within the analysis window. In addition, because the input scalograms were resized to 224 × 224 pixels and Grad-CAM was computed from the last convolutional layer of ResNet-18, the underlying activation maps had a coarse spatial resolution of 7 × 7. Therefore, precise localization of specific frequency regions should be interpreted with caution.

However, Grad-CAM identifies image regions that contribute to a model’s prediction and does not directly establish the physiological mechanisms underlying those regions. Because muscle fiber composition, motor unit conduction properties, and histological changes were not directly measured in the present study, the observed Grad-CAM patterns cannot be interpreted as direct evidence of Type II muscle fiber atrophy or any specific physiological mechanism. Further studies combining sEMG-based deep learning with direct physiological measurements are required to determine the biological basis of these model attention patterns.

Previous sEMG studies have reported age-associated differences in neuromuscular and spectral characteristics, although their outcome measures are not directly comparable with the classification metrics reported here. Guo et al. [12] investigated vastus lateralis motor units in 15 younger and 15 older men using high-density sEMG and reported lower estimates of persistent inward currents in the older group. Specifically, the estimated ΔF values were 3.11 ± 0.77 and 1.86 ± 0.65 pulses/s in the younger and older groups, respectively (*p* = 0.006). Ebenbichler et al. [17] analyzed back-muscle sEMG fatigue-related measures in 114 younger and 108 older individuals with chronic low back pain, using instantaneous median-frequency-based indices during repeated back-extension tasks. Their findings demonstrated age-associated differences in electromyographic fatigue-related characteristics. These studies investigated electrophysiological and spectral measures rather than age-group classification using CWT scalograms.

In the present study, the CWT-ResNet-18 model correctly classified 560 of 709 test image windows, corresponding to an accuracy of 78.98% and a ROC-AUC of 0.8589. These findings indicate that the model could discriminate between CWT image windows originating from young and older adults under the applied random image-window-level split. However, the findings do not establish reliable classification of previously unseen participants or demonstrate that the discriminative patterns specifically reflect age-related neuromuscular alterations. Furthermore, overlapping windows from the same participant could appear in both training and test subsets, and the test subset was also used for checkpoint selection, potentially leading to optimistic performance estimates. Direct quantitative comparison with previous studies is limited by differences in participant characteristics, muscle sites, experimental tasks, outcome measures, and evaluation procedures. Participant-independent validation and direct physiological measurements are required before stronger conclusions can be drawn.

### 4.2. Potential Clinical Implications and Future Applications

The findings of this study demonstrate the preliminary feasibility of combining sEMG-based time–frequency analysis with deep learning to classify recordings obtained from young and older adults. However, the observed classification performance cannot be attributed specifically to age-related neuromuscular characteristics because differences in movement timing, active and resting periods, and anthropometric characteristics were not independently controlled. Because sEMG can be acquired non-invasively from the skin surface, the proposed framework may provide a methodological basis for future investigations of muscle function. Further validation under participant-independent conditions, with appropriate control of task execution and body size, is necessary before the model can be considered a tool for characterizing age-related neuromuscular changes.

The applicability of this framework to real-world wearable sEMG monitoring remains uncertain. Unlike recordings obtained under controlled experimental conditions, wearable measurements may be affected by electrode displacement, variations in skin–electrode contact, motion artifacts, and changes in electrode placement between recording sessions. These factors can alter signal amplitude, spectral characteristics, and the resulting CWT scalograms, potentially reducing classification performance when the model is applied to recordings acquired outside the original experimental setting. Because these sources of variability were not systematically evaluated in the present study, the robustness of the proposed model to wearable recording conditions has not been established. Future studies should investigate electrode placement variability, movement-related artifacts, and between-session reproducibility using independent recordings obtained under realistic wearable conditions.

However, the present model was developed to distinguish between healthy young and older adults based on chronological age and was not trained or validated using clinically diagnosed sarcopenia. Therefore, the current results should not be interpreted as demonstrating the ability to diagnose or screen for sarcopenia. Similarly, fall risk and rehabilitation outcomes were not directly evaluated in this study.

Future studies should investigate whether the proposed sEMG-based framework can distinguish individuals with clinically confirmed sarcopenia from healthy older adults and should compare model predictions with established clinical measures of muscle health, including muscle strength, physical performance, and muscle mass [25]. Validation in larger and more diverse clinical populations will be necessary before the proposed approach can be considered for clinical screening or monitoring applications.

Because chronological age is already known, the ability to distinguish sEMG recordings from young and older adults does not, by itself, establish additional clinical utility. Although grip strength, gait speed, and SARC-F scores were collected to characterize participants in the older-adult group, these measures were not included in model development or analyzed in relation to model predictions. Therefore, the present findings do not establish whether the model provides clinically meaningful information about muscle function beyond chronological age. Future studies should investigate associations between participant-level model predictions and these clinical measures, with appropriate adjustment for chronological age and validation using participant-independent data, to determine the potential incremental clinical value of the proposed framework.

### 4.3. Limitations and Future Work

Importantly, the purpose of the present model is not to predict chronological age when age information is already available. Rather, this study was designed as a proof-of-concept investigation to determine whether sEMG time–frequency representations contain signal patterns that systematically differ between younger and older adults. Therefore, the value of the model lies in investigating the presence of age-group-associated information in sEMG signals and exploring the time–frequency regions contributing to classification, rather than providing information beyond chronological age itself.

This study has several limitations. First, the participants consisted of relatively healthy young and older adults without musculoskeletal or neurological disorders, and the groups were defined according to chronological age rather than clinical sarcopenia status. Therefore, the classification performance observed in this study represents discrimination between age groups and should not be interpreted as diagnostic performance for sarcopenia.

Second, the study included a relatively limited number of participants, with 30 young and 30 older adults. The original evaluation employed a single random train–test split at the CWT image-window level rather than participant-independent data partitioning, and no separate validation set was used. Consequently, multiple windows from the same participant could appear in both training and test subsets, potentially allowing the model to learn participant-specific characteristics rather than age-group-associated signal patterns alone. Furthermore, the use of 50% overlapping windows and bilateral recordings introduced additional dependence among samples. These factors may have resulted in optimistic performance estimates. In addition, the model checkpoint was selected based on the highest test accuracy across 20 training epochs, without a separate validation dataset. The selected checkpoint was obtained after the 19th epoch, which achieved a test accuracy of 78.98%. Because the same test subset was used for both checkpoint selection and final performance evaluation, the reported window-level classification metrics may be further affected by optimistic bias. Future studies should use an independent validation subset for checkpoint selection and reserve the test subset exclusively for final evaluation.

The reported evaluation was based on 709 test windows, including 314 windows from older adults and 395 from young adults, rather than independent participants. The number of independent participants represented in the test set was not determined, and the imbalance in window counts may have influenced the reported accuracy. Therefore, these classification metrics should not be interpreted as evidence of generalization to previously unseen participants. Future studies should employ participant-grouped cross-validation, such as grouped k-fold or leave-one-subject-out cross-validation, repeated across multiple random seeds where applicable, with hyperparameter selection restricted to the training folds. Participant-level predictions should be obtained by aggregating window-level prediction probabilities, and corresponding 95% confidence intervals should be estimated using participant-level resampling. In addition, recruitment from a single university setting may limit the representativeness of the study population and the generalizability of the findings to other populations and recording environments.

Furthermore, the uneven distribution of CWT image windows between the two age groups raises concerns regarding dataset composition and traceability. Differences in window counts may reflect variation in recording duration, task execution, segmentation, or data availability. Because these factors were not independently evaluated, their potential influence on classification performance cannot be excluded. A participant-level audit of recording duration and window counts by muscle site and recording side is necessary to establish dataset traceability and clarify the origin of the observed class imbalance.

Third, the sEMG signals were collected during predefined muscle-specific movements, but movement cadence and contraction timing were not externally controlled, and no explicit activity-onset or offset detection was applied before segmentation. Consequently, individual windows could contain different proportions of active contraction, transition periods, and relative rest. Temporal characteristics such as movement rhythm and duty cycle remained available to the model and may have contributed to classification performance. A timing-only negative-control model and analyses restricted to activity-matched segments were not performed in the present proof-of-concept study. Therefore, the present results cannot distinguish age-related neuromuscular signal characteristics from potential differences in task timing or movement pattern. Future studies should incorporate activity-based segmentation, standardized or matched duty cycles, and timing-only control models to determine the extent to which temporal task characteristics contribute to classification. In addition, contraction force was not quantitatively measured or normalized across participants during the recording tasks. Therefore, differences in contraction intensity may have influenced the recorded sEMG patterns and contributed to the observed classification performance. Future studies should incorporate standardized force targets or force-normalized recording protocols where appropriate.

Fourth, the young and older groups differed significantly in height (*p* < 0.001), whereas body weight (*p* = 0.260) and BMI (*p* = 0.376) did not differ significantly between groups. Because anthropometric characteristics and subcutaneous tissue thickness can influence surface EMG signal characteristics, differences in body size may have contributed to the observed classification performance. Subcutaneous tissue thickness was not directly measured in this study; therefore, its potential confounding effect could not be quantified. Future studies should consider controlling for anthropometric characteristics and directly assessing subcutaneous tissue thickness when evaluating age-related differences in sEMG signals.

Fifth, the physiological mechanisms underlying the discriminative time–frequency patterns identified by the model were not directly measured. Although Grad-CAM provided information regarding the regions of the CWT scalograms that contributed to model predictions, muscle fiber composition, motor unit characteristics, and other physiological factors were not independently assessed. Prior work has documented regional recruitment and differential motor unit behavior during postural control in older adults [26]. Therefore, the Grad-CAM results should be interpreted as model-level explanatory evidence rather than direct physiological evidence.

Sixth, the proposed CWT-ResNet approach was not directly compared with conventional feature-based classifiers, one-dimensional CNN models, or alternative time–frequency representations. Therefore, the present findings do not establish whether the combination of CWT and ResNet-18 provides an improvement over simpler classification methods or alternative signal representations. In particular, the relative contributions of time–frequency transformation and deep-learning architecture to the observed classification performance remain undetermined. Future studies should compare conventional amplitudes and spectral features, including the root mean square (RMS), mean frequency (MNF), median frequency (MDF), and frequency-band power, using logistic regression and support vector machine (SVM) classifiers. Additional comparisons should include one-dimensional CNNs trained on raw sEMG windows and ResNet-18 models trained on short-time Fourier transform (STFT) spectrograms. All approaches should be evaluated using identical participant-grouped cross-validation folds, consistent preprocessing procedures, and comparable performance metrics to determine whether CWT-ResNet-18 provides measurable advantages over these baseline methods.

Future studies should include larger and more diverse populations, particularly older adults with clinically confirmed sarcopenia, and compare sEMG-based model predictions with established clinical measures of muscle strength, physical performance, and muscle mass [27]. Previous research has also examined surface EMG biomarkers of sarcopenic motor dysfunction during postural stabilization [28]. Additional validation using independent datasets and multimodal physiological measurements may further clarify the clinical relevance and biological basis of the sEMG patterns identified by the proposed framework. Although grip strength and gait speed were assessed in the older-adult group, their associations with the model’s predicted probabilities were not evaluated. Therefore, the present study cannot establish whether the model outputs reflect clinically meaningful variation in muscle function beyond chronological age. Future studies should examine these relationships using participant-level predictions and include direct assessments of muscle mass where available.

## 5. Conclusions

This study investigated a deep-learning-based framework for distinguishing sEMG recordings obtained from young and older adults using CWT-based time–frequency representations and ResNet-18. The proposed model achieved a window-level classification accuracy of 78.98% (560/709) and a ROC-AUC of 0.8589 under a random image-window-level train–test split. Because the partitioning was performed at the image-window level, overlapping windows from the same participant could appear in both training and test subsets, potentially allowing the model to learn participant-specific characteristics and leading to optimistic performance estimates. Therefore, these results should not be interpreted as evidence of generalization to previously unseen participants.

Muscle-specific evaluation was performed using subsets of test windows from the medial gastrocnemius, rectus femoris, and erector spinae within a single model trained on pooled muscle data. The observed classification accuracies were 78.81%, 78.57%, and 79.89%, respectively. These descriptive results do not establish meaningful performance differences between muscle sites. Grad-CAM provided illustrative visualizations of image regions contributing to individual model predictions but did not establish systematic age-group differences in attention patterns or validate specific physiological mechanisms.

Overall, the findings demonstrate preliminary feasibility of applying CWT-based time–frequency representations and deep learning to distinguish sEMG windows from young and older adults under the present experimental conditions. The model was not designed or validated to diagnose sarcopenia or to provide clinical information beyond chronological age. Participant-independent cross-validation, together with participant-level performance estimates and confidence intervals, is necessary to establish the generalizability of the proposed framework. Further research comparing the proposed approach with simpler baseline models and examining associations with independent measures of muscle function is also required to determine its clinical relevance.

## Figures and Tables

**Table 1:** Distribution of source recordings and CWT windows by age group, muscle site, and recording side.

Muscle Site	Side	Young Files	Young Windows	Older Files	Older Windows
Medial gastrocnemius	Left	30	341	30	287
Medial gastrocnemius	Right	30	329	30	281
Rectus femoris	Left	30	362	30	310
Rectus femoris	Right	30	397	30	372
Erector spinae	Not specified	30	456	30	408
Total	-	150	1885	150	1658

**Table 2:** Summary of general characteristics of the study population and sEMG dataset.

Category	Item	Young Group	Older Group	*p*-Value
Demographics	Age, years	24.5 ± 2.3	72.1 ± 4.5	-
	Sex, male/ female	15/15	14/16	-
	Height, cm	170.97 ± 8.34	161.50 ± 5.99	<0.001
	Weight, kg	69.54 ± 18.90	64.82 ± 12.55	0.260
	BMI, kg/m^2^	23.66 ± 5.63	24.82 ± 4.33	0.376
Physical Function and Screening	Grip strength, left (kg)	37.60 ± 12.13	25.34 ± 9.34	-
	Grip strength, right (kg)	39.04 ± 11.38	25.48 ± 8.14	-
	Gait speed (m/s)	0.98 ± 0.16	1.03 ± 0.16	-
	SARC-F score	0.97 ± 1.25	1.23 ± 1.38	-
Subjects by Muscle	Medial gastrocnemius	30	30	-
	Rectus femoris	30	30	-
	Erector spinae	30	30	-
Window Datasets	Test, *n*	395	314	-
	Test proportion	55.71%	44.29%	-

**Table 3:** Muscle-specific classification performance of the proposed CWT-ResNet model.

Muscle Site	Accuracy	Young, *n*	Older, *n*	Test Windows, *n*
Medial gastrocnemius	78.81%	144	125	269
Rectus femoris	78.57%	157	109	266
Erector spinae	79.89%	94	80	174
Total	78.98%	395	314	709

## Data Availability

The data generated and analyzed in this study are not publicly available due to participant privacy and ethical considerations.
